# Project ESTEEM protocol: a randomized controlled trial of an LGBTQ-affirmative treatment for young adult sexual minority men’s mental and sexual health

**DOI:** 10.1186/s12889-019-7346-4

**Published:** 2019-08-09

**Authors:** John E. Pachankis, Erin M. McConocha, Jesse S. Reynolds, Roxanne Winston, Oluwaseyi Adeyinka, Audrey Harkness, Charles L. Burton, Kriti Behari, Timothy J. Sullivan, Adam I. Eldahan, Denise A. Esserman, Mark L. Hatzenbuehler, Steven A. Safren

**Affiliations:** 1 Department of Social and Behavioral Sciences, Yale School of Public Health, New Haven, USA; 2 Department of Biostatistics, Yale School of Public Health, New Haven, USA; 3 Department of Public Health Sciences, University of Miami, Miami, USA; 4 Department of Sociomedical Sciences, Columbia University Mailman School of Public Health, New York City, USA; 5 Department of Psychology, University of Miami, Miami, USA

## Abstract

**Background:**

Young gay and bisexual men disproportionately experience depression, anxiety, and substance use problems and are among the highest risk group for HIV infection in the U.S. Diverse methods locate the source of these health disparities in young gay and bisexual men’s exposure to minority stress. In fact, minority stress, psychiatric morbidity, substance use, and HIV risk fuel each other, forming a synergistic threat to young gay and bisexual men’s health. Yet no known intervention addresses minority stress to improve mental health, substance use problems, or their joint impact on HIV risk in this population. This paper describes the design of a study to test the efficacy of such an intervention, called ESTEEM (Effective Skills to Empower Effective Men), a 10-session skills-building intervention designed to reduce young gay and bisexual men’s co-occurring health risks by addressing the underlying cognitive, affective, and behavioral pathways through which minority stress impairs health.

**Methods:**

This study, funded by the National Institute of Mental Health, is a three-arm randomized controlled trial to examine (1) the efficacy of ESTEEM compared to community mental health treatment and HIV counseling and testing and (2) whether ESTEEM works through its hypothesized cognitive, affective, and behavioral minority stress processes. Our primary outcome, measured 8 months after baseline, is condomless anal sex in the absence of PrEP or known undetectable viral load of HIV+ primary partners. Secondary outcomes include depression, anxiety, substance use, sexual compulsivity, and PrEP uptake, also measured 8 months after baseline.

**Discussion:**

Delivering specific stand-alone treatments for specific mental, behavioral, and sexual health problems represents the current state of evidence-based practice. However, dissemination and implementation of this one treatment-one problem approach has not been ideal. A single intervention that reduces young gay and bisexual men’s depression, anxiety, substance use, and HIV risk by reducing the common minority stress pathways across these problems would represent an efficient, cost-effective alternative to currently isolated approaches, and holds great promise for reducing sexual orientation health disparities among young men.

**Trial registration:**

Registered October 10, 2016 to ClinicalTrials.gov Identifier: NCT02929069.

## Background

Young gay and bisexual men are at disproportionate risk of depression, anxiety, and substance use problems [1, 2], which synergistically fuels their increasing risk of HIV infection [3, 4]. Male sexual orientation-related mental health and substance disparities arise early in development and persist across the life course [2]. At the same time, young gay and bisexual men represent one of the most severely at-risk groups for new HIV infection [5]. Among young men, approximately 93% of all diagnosed HIV infections are male-to-male [6]. Mental health problems can influence young gay and bisexual men’s HIV risk. For instance, depression can impede the initiation and maintenance of health behaviors and, in turn, can increase HIV risk [7–10]. Anxiety might increase HIV-risk behavior through avoidant coping and disengagement [11]. Social anxiety predicts poor condom use communication and actual condom use [12, 13]. Gay and bisexual men with PTSD report a nearly three-fold greater likelihood of recent condomless anal sex than those without PTSD [14]. Thus, mental health and substance use disparities drive young gay and bisexual men's HIV- risk behavior [3, 15–18].

Despite HIV risk being influenced by mental health disparities for young gay and bisexual men, no evidence-based mental health intervention specifically tailored to this population exists. Young adulthood (ages 18–35) for young gay and bisexual men represents a developmental period of particularly high identity-related stress and therefore an important opportunity for intervention [19]. Identity-affirming interventions during this period can potentially avert the onset of mental illness and substance abuse, and prevent co-occurring risk of HIV infection [18]. Yet, no identity-affirming intervention has been tested for efficacy for improving young gay and bisexual men’s mental health, despite the fact that young gay and bisexual men represent one of the highest-risk groups for depression, anxiety, substance use, and HIV infection. Young gay and bisexual men are more likely to seek mental health services compared to heterosexuals [20], making the absence of evidence-based mental health treatments for this population even more striking. This gap may be explained by the fact that, until recently, no clear model existed for explaining and addressing the unique determinants of young gay and bisexual men’s elevated mental health impairment that drives their risk of HIV infection.

Growing evidence suggests that young gay and bisexual men’s co-occurring mental health, substance use, and HIV risks are rooted in early and ongoing stigma-related stress, known as minority stress. The ultimate source of minority stress is *structural stigma*, or the societal structures that deny young gay and bisexual men the same rights and opportunities afforded heterosexuals [21]. Structural stigma encourages discrimination within families, religious communities, schools, workplaces, and everyday interactions, elevating young gay and bisexual men’s stress, and therefore psychiatric burden [21, 22]. Peer teasing and bullying [23], subtle and overt forms of parental rejection [24–26], and feelings of difference [27] set the stage for mental illness, substance use, and sexual risk-taking into young adulthood and beyond. The first decade after coming out is the most strongly associated with mental illness among young gay and bisexual men [28].

**Fig. 1:**
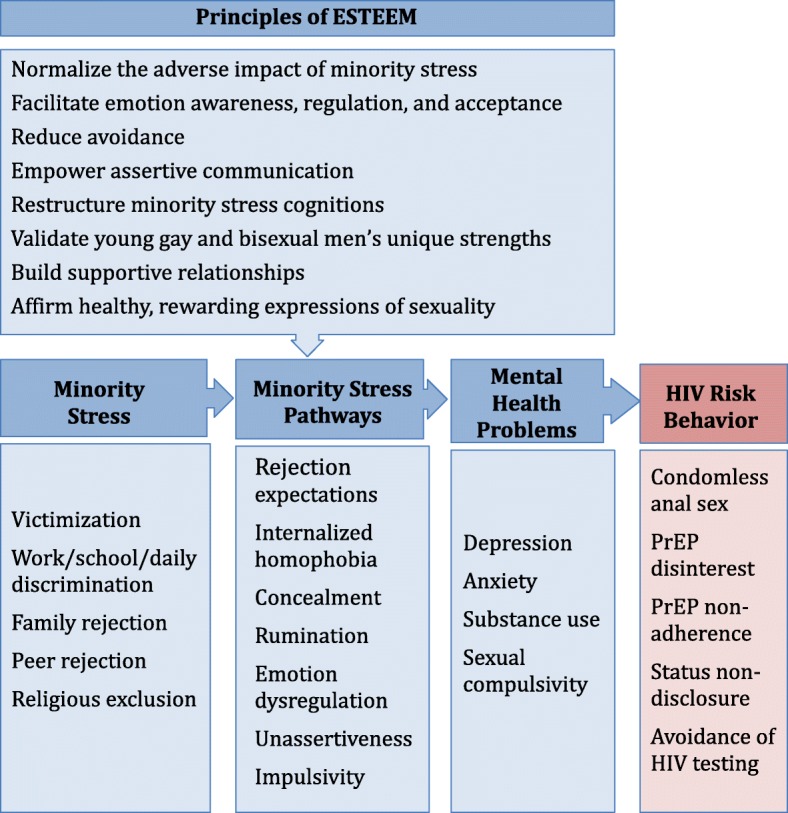
Conceptual model

Cognitive, affective, and behavioral stress pathways emerging from minority stress offer clear targets for improving mental health and reducing HIV risk among young gay and bisexual men (see Fig. 1). Minority stress theory suggests that stigma compromises young gay and bisexual men’s health through several psychosocial processes [29, 30]. Some of these processes are cognitive and affective in nature, such as internalized homophobia and rejection schemas [31, 32]. Others are characterized by behavioral avoidance, such as sexual orientation concealment, unassertiveness, and impulsivity [33]. Some of these processes, like internalized homophobia, are specific to sexual minorities [32, 34]; others are universal risk factors for psychopathology that are elevated among young gay and bisexual men, such as emotion dysregulation and low self-worth [35, 36]. Strong and growing evidence suggests that each of young gay and bisexual men’s co-occurring health risks is rooted in these minority stress pathways [37]. Depression, anxiety, and condomless anal sex are associated with gay-related rejection expectations, internalized homophobia, and concealment [32, 34, 38–41]. Substance use and sexual compulsivity are associated with rejection expectations and internalized homophobia [42, 43]. HIV risk is predicted by low self-worth, unassertiveness, and impulsivity [12, 27, 44]. These cognitive, affective, and behavioral pathways, in turn, mediate the relationship between minority stress and mental health and HIV risk and represent promising treatment targets [39, 40].

The model guiding our intervention (Fig. 1) suggests that reducing the cognitive, affective, and behavioral pathways associated with minority stress can simultaneously improve young gay and bisexual men’s co-occurring mental, behavioral, and sexual health risks [30, 37]. Drawing on advancements in emotion science [45], psychiatric nosology [46], and cognitive-affective neuroscience [47], evidence suggests that young gay and bisexual men’s psychosocial health risks (e.g., depression, substance use, condomless anal sex) are functionally similar when seen to represent maladaptive reactions to minority stress [37]. For example, chronic exposure to the types of stressors that young gay and bisexual men disproportionately face from an early age disrupts neurobiological stress pathways and yields rejection schemas, emotion dysregulation, unassertiveness, and impulsivity [48]. Given that these pathways underlie young gay and bisexual men’s co-occurring depression, anxiety, substance use, and HIV -risk behavior, a single treatment that reduces them can efficiently improve young gay and bisexual men’s multiple health problems all at once.

Young gay and bisexual men’s health interventions currently use a one-problem/one-treatment approach. Some interventions promote condom use [49], some encourage PrEP initiation and maintenance [50, 51], and others reduce substance use [52]. These treatments show moderate efficacy [53]. None currently seeks to reduce mental health problems (e.g., depression, anxiety) among at-risk young gay and bisexual men. A unified, transdiagnostic approach that addresses the pathways that unite these conditions may increase effectiveness, reduce cost, and provide a streamlined treatment experience for the most vulnerable young gay and bisexual men, who are unlikely to seek multiple treatments for multiple health concerns.

### Study objectives

The first objective of this study is to test the efficacy of a 10-session skills-building intervention designed to reduce young gay and bisexual men’s co-occurring health risks by addressing the underlying cognitive, affective, and behavioral pathways through which minority stress impairs health. This study will test the efficacy of this treatment, called ESTEEM (Effective Skills to Empower Effective Men), against (1) community mental health treatment (CMHT) and (2) HIV voluntary counseling and testing (VCT) only. Knowing whether ESTEEM yields greater improvement than time-matched CMHT will establish the benefit of ESTEEM’s transdiagnostic approach. Comparing ESTEEM to VCT-only offers a test of ESTEEM’s incremental efficacy. Outcomes across conditions will be primarily compared at the 8-month follow-up given that these cognitive, affective, and behavioral changes take time to take root; longitudinal modeling will examine change across all time points (i.e., baseline, 4-, 8-, and 12-month follow-ups).

The second objective of this study is to determine whether ESTEEM works through its hypothesized cognitive, affective, and behavioral minority stress processes. 4-, 8-, and 12-month follow-ups will allow studying whether improvements in minority stress processes precede and statistically mediate outcome improvements. Mediation will validate the minority stress theory of ESTEEM and provide transdiagnostic targets for future health interventions for young gay and bisexual men.

## Methods/Design

### Design

**Fig. 2:**
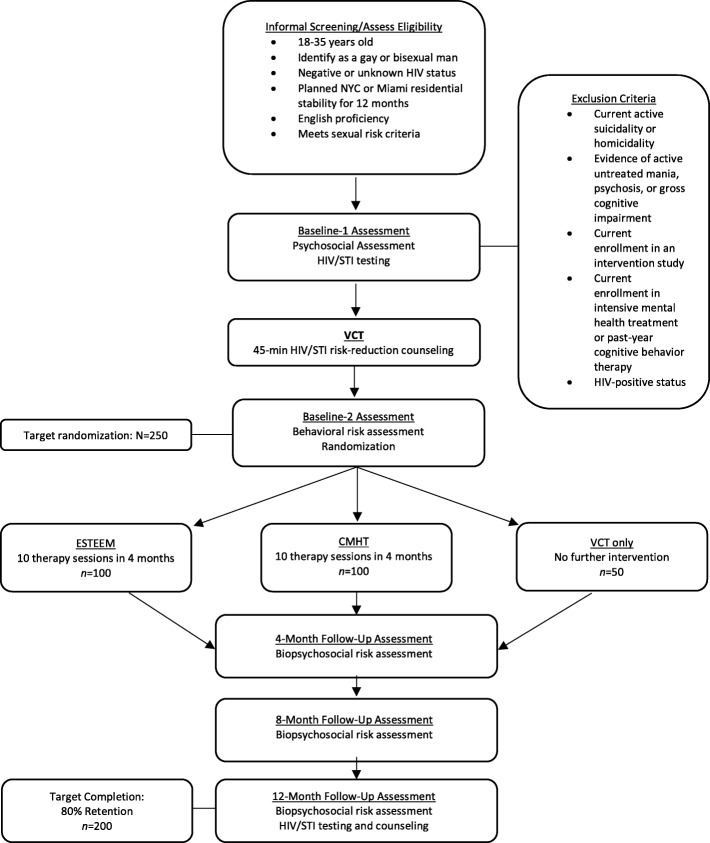
Flow chart of study procedures

This study utilizes a three-arm randomized controlled trial design, in New York City and Miami, to test the efficacy of ESTEEM (see Fig. 2). We will utilize two comparison conditions—LGBTQ-affirmative community mental health treatment (CMHT) and brief voluntary HIV counseling and testing (VCT) only. Follow-up assessments at 4-, 8-, and 12-months will allow us to test whether changes in minority stress and mental health precede and statistically mediate the efficacy of ESTEEM.

### Recruitment

We are using active and passive recruitment strategies. Active approaches involve conducting eligibility screening via electronic tablet at bars/clubs, support groups, and community events (e.g., groups at the NYC LGBT Center, LGBTQ pride events in NYC and Miami). Passive approaches involve advertising on young gay and bisexual men-oriented mobile apps and websites (e.g., Grindr, Scruff, BGCLive, Growlr), clinic waiting rooms, social media (e.g., Craigslist, Facebook, Reddit, party listservs), and referrals from previous or current study participants. As part of passive recruitment, we also contact participants from previous research studies who consented to be contacted for future studies. Our advertisements engage help-seeking young gay and bisexual men by emphasizing the study as a safe venue for discussing mental health and sexuality.

### Eligibility

#### Inclusion criteria

Eligible intervention participants meet the following criteria: (1) aged 18–35, (2) identify as a gay or bisexual man, (3) HIV-negative status confirmed through in-office testing, (4) diagnosis of any *DSM* depressive, anxiety, or trauma- and stressor-related disorder; (5) HIV sexual risk (≥1 act of past-90-day condomless anal with a male partner of unknown status or HIV+ status, unless with a HIV+ primary/main partner with known undetectable viral load); (6) not currently adherent to PrEP (defined as taking 4 or more days per week) (7) NYC or Miami residential stability and planned availability for 12 months; (8) English-language proficiency; and (9) provision of informed consent.

#### Exclusion criteria

Intervention participants are excluded for any of the following: 1) current active suicidality or homicidality (defined as active intent or concrete plan, as opposed to passive suicidal ideation); 2) evidence of active untreated mania, psychosis, or gross cognitive impairment; 3) current enrollment in an intervention study; 4) current enrollment in intensive mental health treatment (receiving treatment more than once per month or 8 or more sessions of cognitive-behavioral therapy (CBT) within the past year); or 5) HIV-positive status.

### Randomization

Participants are randomly assigned to receive one of the following three conditions: ESTEEM, CMHT, or VCT-only (*N* = 250 across both sites). We are using a 2:2:1 randomization scheme such that for every two participants randomized to ESTEEM, two participants will be randomized to CMHT and one participant will be randomized to VCT-only. Randomization happens through a computer-generated program across both study sites, such that 150 will be randomized at the NYC site (60 ESTEEM, 60 CMHT, 30 VCT-only) and 100 will be randomized at the Miami site (40 ESTEEM, 40 CMHT, 20 VCT-only).

### Description of ESTEEM condition

ESTEEM is a 10-session intervention based on the Unified Protocol [54], an individually-delivered CBT intervention with efficacy for reducing stress-sensitive mental health disorders (e.g., depression, anxiety) by enhancing emotion regulation skills; reducing avoidance patterns; and improving motivation and self-efficacy for behavior change [54, 55]. The Unified Protocol employs modules for motivation enhancement, interoceptive and situational exposure, cognitive restructuring, mindfulness, and self-monitoring techniques. Through an extensive adaptation process [56], we adapted the Unified Protocol to enhance young gay and bisexual men’s stigma coping by reducing minority stress processes (see Table 1). For example, modules were adapted to help young gay and bisexual men identify minority stress experiences; track unhealthy reactions to minority stress, focusing on avoidance reactions, like substance use and condomless anal sex; attribute distress to minority stress rather than to personal failure; and assert themselves against unjust minority stress in safe situations. Adaptations were infused throughout the Unified Protocol *Therapist Workbook* [57]; this adaptation served as the therapist manual. ESTEEM participants complete 10 sessions of therapy, with one session per week. If participants miss sessions or need to reschedule, we will make every effort to reschedule sessions such that participants stay as close to a one session per week schedule. If participants miss a week, they may be rescheduled to do two sessions in 1 week, but they will be told the goal is once per week. All sessions must be completed within 4 months.

**Table 1:** Content of ESTEEM intervention

Module 1: Motivation Enhancement for ESTEEM Engagement	
Discuss HIV test result and provide PrEP information and referral Review individualized health report against young gay and bisexual men norms Clarify primary mental, behavioral, and sexual health goals Build motivation to address mental, behavioral, and sexual health Review unique strengths as a young gay and bisexual men	
Module 2: The Nature and Emotional Impact of Minority Stress	
Review the impact of minority stress on mental and sexual health Identify specific early and ongoing forms of minority stress Discuss current coping strategies	
Module 3: Tracking Emotional Experiences	
Raise awareness of the emotional impact of early and ongoing minority stress	
Module 4: Awareness of Minority Stress Reactions	
Raise awareness of the behavioral impact of minority stress Teach mindful, present-focused reactions to minority stress	
Module 5: Cognitive Appraisal and Reappraisal	
Connect minority stress to maladaptive thinking patterns Identity thoughts driven by minority stress and learn to update them	
Module 6: Emotion Avoidance	
Learn how avoiding strong emotions can lead to unhealthy behavior Discuss how minority stress might lead to avoidance of certain people, places, or experiences	
Module 7: Emotion-Driven Behaviors	
Focus on the ways that minority stress can lead to avoidance Discuss intimacy, relationships, and substance use	
Module 8: Behavioral Skills Training	
Explore how minority stress can lead to unassertiveness Focus on assertiveness training for coping with minority stress	
Module 9: Behavioral Experiments	
Create an emotional and behavioral avoidance hierarchy Engage young gay and bisexual men in behavioral experiments in which previously avoided experiences are gradually confronted	
Module 10: Relapse Prevention	
Review new cognitive, affective, and behavioral coping strategies and their application to future minority stress experiences	

### Description of community mental health treatment condition

The current standard of care for LGBTQ individuals who seek mental, behavioral, or sexual health care is LGBTQ-affirmative therapy [58]. The practice of LGBTQ-affirmative therapy is outlined across 21 guidelines published by the American Psychological Association. However, the efficacy of LGBTQ-affirmative psychotherapy has never been tested [59], despite several promising case studies [60–62]. We refer young gay and bisexual men to community clinicians who provide this standard of care. These community clinicians are located in community clinics providing LGBTQ-affirmative psychotherapy, one in New York City, one in Miami. Similar to participants randomized to ESTEEM, CMHT participants will complete 10 sessions of therapy, with one session per week. If participants miss sessions or need to reschedule, we make every effort to reschedule sessions such that participants stay as close to a one session per week schedule. If participants miss a week, they may be rescheduled to do two sessions in 1 week, but they are told the goal is once per week. All sessions must be completed within 4 months.

### Description of voluntary HIV counseling and testing

Participants in all arms receive VCT-only at their first baseline visit, before being randomized to their respective arms—ESTEEM, CMHT, or VCT-only—and again at the 12-month follow-up visit. Participants randomized to the VCT-only arm do not receive any further intervention. We base VCT on CDC guidelines and the control arms of large community-based RCTs (e.g., Projects RESPECT, EXPLORE, AWARE) [63–66]. VCT-only consists of one unique 45-min session given that 1-session VCT is as effective as 2-session VCT for gay and bisexual men [65]. At the beginning of the session, the counselor explains the purpose of HIV and STI testing, and with the participant’s consent, administers an OraQuick® Rapid HIV-1/2 antibody test. While waiting for test results, the participant provides a urine sample and oral and rectal swabs for chlamydia and gonorrhea testing, and, when done, the counselor reviews a handout containing facts about HIV/STI transmission risk and PrEP. Misconceptions are clarified. This handout contains provider referrals for participants interested in PrEP or other HIV/STI prevention services. The counselor then engages in a person-centered discussion to elicit the participant’s current risk behavior, the contextual drivers of the behavior, perceptions of the pros/cons of continuing the behavior, and self-efficacy for changing the behavior based on the participant’s past success. A personalized risk-reduction plan is created that includes specific, achievable goals the participant can implement to reduce risk [65]. These goals are written on the risk-reduction plan handout. Participants who receive a preliminary positive HIV test result are referred to their current medical provider or nearby community health centers for confirmatory testing and appropriate care. Urine, oral, and rectal samples are sent to a lab (Quest Diagnostics) for testing. We notify participants who screen positive for chlamydia or gonorrhea immediately upon receiving the lab results and refer them to a nearby community health center or their own medical provider for treatment.

### Study assessments

Participants provide the following data at baseline and 4-, 8-, and 12-month follow-up appointments, administered at home and in-office: (1) interviewer-administered assessment of HIV-risk behavior, including condomless anal sex, including while under the influence of drugs or alcohol; PrEP use and adherence; and number of sexual partners, during the previous 3 months; (2) interviewer-administered and self-report mental health assessment; and (3) self-report assessments of minority stress pathways At baseline and 12-month appointments,participants complete biological assessments of HIV, chlamydia, and gonorrhea infection. After each intervention sessions, participants randomized to the ESTEEM or CMHT condition complete a post-session assessment of intervention engagement.

### Primary outcome

#### HIV-risk behavior

Our primary outcome is condomless anal sex in the absence of either PrEP or known undetectable viral load of HIV+ primary partners, measured with the Time-Line Follow-Back (TLFB), a semi-structured interview [67]. The TLFB will yield past-90-day incidence of HIV risk behavior: condomless anal sex, sex while using drugs or alcohol, number of sexual partners, and preceding-week PrEP use (i.e., coverage defined as 4+ doses per week). TLFB interviewers will be masked to study arm.

### Secondary outcomes

#### Mental health

The Mini-International Neuropsychiatric Interview (MINI), a structured psychiatric interview [68] allows non-clinician research assistants (RAs) to derive *DSM* and *ICD* psychiatric diagnoses. To determine symptom severity, interviewers also administer the Hamilton Rating Scale for Depression (HAM-D) [69]. Interviewers using the MINI and HAM-D are masked to study arm.

Participants complete the Brief Symptom Inventory (BSI) [70], the Center for Epidemiology Studies Depression Scale (CES-D) [71], the Beck Anxiety Inventory [72, 73], the Overall Anxiety and Depression Severity and Impairment Scales [74], the Social Interaction Anxiety Scale [57, 75], and the Sexual Compulsivity Scale [76]. In addition to the MINI substance use module, participants complete the self-report Short Inventory of Problems-Alcohol and Drugs (SIP-AD), capturing negative consequences of substance use across life domains [77].

### Minority stress pathways

We assess the minority stress pathways proposed to underlie our intervention (Fig. 1) with reliable/valid measures of: gay-related rejection sensitivity [31], internalized homonegativity [78], sexual orientation concealment [79], sexual minority identity development and conflict [80], difficulties of emotion regulation [81], rumination [82], impulsiveness [83], and assertiveness [84].

#### HIV, chlamydia, and gonorrhea infection

At baseline and 12-month follow-up, we use Orasure Rapid HIV-1/2 antibody test and collect urine samples and rectal and oral swabs for chlamydia and gonorrhea testing.

#### Intervention engagement

ESTEEM and CMHT sessions are video- or audio-taped in the settings where they are delivered to monitor intervention fidelity for the ESTEEM sessions and potential contamination with ESTEEM elements in the CMHT condition. Also, both therapists and participants complete short surveys after each therapy session at the appointment site regarding perceptions of treatment; ESTEEM participants complete a short comprehension quiz to assess engagement.

### Data analyses

#### Sample size justification

Our primary goal is to demonstrate a greater reduction at 8 months in condomless anal sex in the absence of either PrEP or known undetectable viral load of HIV+ primary partners, in the ESTEEM arm versus the CMHT and VCT-only arms. In our pilot study, we saw a 60% reduction in condomless anal sex at 6 months in the ESTEEM arm [85]. We used these estimates to inform our 8-month endpoint. Based on previous studies of VCT [64] and the fact that CMHT does not specifically focus on condomless anal sex, we expect that these arms will yield lower reductions compared to ESTEEM, but a slightly larger reduction in CMHT (20%) compared to VCT-only (15%). To achieve at least 90% power at a 5% type I error rate, accounting for an R-square of 0.1 between treatment arm and the covariates (e.g., site, race/ethnicity), we will need 80 individuals in the ESTEEM and CMHT arms, and 40 individuals in the VCT-only arm. Although we plan to take steps to increase our retention rate from our pilot study, we conservatively estimate the retention at 8 months to be 80%. Therefore, we plan to randomize 100 ESTEEM,100 CMHT, and 50 VCT-only young gay and bisexual men. Sample size calculations were carried out using PASS 12 for logistic regression. For secondary outcomes (e.g., mental health, substance use), we will have 80% power to detect an effect size of 0.55 and 0.70 for ESTEEM vs. CMHT and ESTEEM vs. VCT-only, respectively, at a type I error rate of 0.01 (conservative due to multiple testing). These effect sizes are smaller than those found in the pilot.

#### Efficacy analyses

We will use a fixed sequence procedure to control for multiple testing of the primary comparisons: reduction in condomless anal sex in ESTEEM vs. VCT-only and ESTEEM vs. CMHT. We will conduct the comparison of ESTEEM versus VCT-only at the 0.05 level. To make use of all data collected, we will analyze the sex risk outcome using a generalized linear mixed model with a logit link using a contrast to test the comparison at the primary time point of 8 months adjusting for site and race/ethnicity. Secondary outcomes of interest include: the absence of a mental health diagnosis and reduction in mental health illness severity (e.g., depression, anxiety, substance use). We will use generalized linear mixed models (logit link for dichotomous outcomes and identity link for continuous outcomes) adjusting for site and race/ethnicity. If the assumption of normality is violated, we will explore data transformation. To control the false discovery rate, we will use the Benjamini and Hochberg method [86].

#### Mediation analyses

In our mediation analyses, we will examine whether changes in the proposed mediators (e.g. rejection sensitivity, internalized homophobia, emotion dysregulation, unassertiveness) precede and statistically mediate intervention effects consistent with our minority stress model (Fig. 1). We will use path analysis/structural equation modeling to model and assess the size of the indirect effect from intervention condition to 12-month outcomes through mediators assessed at 4- and 8-months controlling for baseline effects of these mediators. Using M*plus* v7.3 [87] to perform a Monte-Carlo simulation power analysis [88], we estimated that sample sizes of 80 (ESTEEM) and 40 (VCT-only), allowing for 20% attrition, would provide at least 80% power to detect a moderate effect size in the indirect treatment effect with a level of significance of 5%, consistent with path sizes in our pilot study [85].

### Ethical research conduct

The study participants are at minimal risk for harm as a result of participation in the proposed research study. Although unlikely, one risk of the proposed study is that participants will experience emotional discomfort while completing the quantitative assessments or the intervention. Breach of participants’ confidentiality presents another possible risk.

It is possible that participants may experience emotional discomfort in responding to assessments or receiving HIV/STI test results. While every possible step will be taken to minimize such risk, consent documentation will make it clear that if participants have any concerns about any aspect of the study they may refuse to continue with the study at any time, without penalty. In addition, we will remind participants during the course of their assessments that they can refuse to answer any questions and may discontinue participation at any time. Staff members at our Yale and Miami sites will be thoroughly trained in appropriate responses to participant distress through ongoing trainings by a licensed clinical psychologist. This training will address the appropriate handling of imminent threats and provision of referrals to free counseling services in less imminent clinical situations. We have developed a protocol for immediately referring participants who learn, as a result of our study, that they are HIV-positive or infected with chlamydia or gonorrhea to a local LGBTQ-affirmative HIV care clinic.

The primary potential risk to participants is breach of confidentiality. Breaches of confidentiality will occur if a participant reports a clear intention to harm himself or another person. Additionally, health care professionals are required by state law to report suspected cases of abuse or neglect. The likelihood that any additional breaches of confidentiality would occur is minimal, as steps will be taken to guard against this risk. To protect participants’ confidentiality, we will obtain an NIH Certificate of Confidentiality prior to enrolling participants. All counselors and RAs will undergo rigorous training in maintaining participants’ privacy and confidentiality and will be in possession of valid Collaborative Institutional Training Initiative (CITI) certificates. Further, immediately upon providing consent, all participants will be assigned an identification number, which will only be kept on an electronic database that will be password protected and located on a secure, password-protected server. This information will not be stored with any other data and no other identifying information will appear on any form. All contact with participants will be made by counselors and research staff under explicit guidelines to preserve confidentiality when telephoning, emailing, or mailing information to participants. All materials with identifying information will be kept in a password-protected electronic file that is separate from participant’s study data. Participants will provide the respective site, Yale or Miami, with alternative contact information (email, phone numbers, and mailing address) for compensation and study retention purposes. This information will be treated in the same confidential manner as all other participant information, as described here.

Given the public health importance addressed by this study and the potential benefit of the information to be gained, we believe that the risk to subjects is reasonable. Sexual-risk behavior among young gay and bisexual men is a clear public health concern. As all participants in the present study will be exposed to information about HIV-transmission risks, we anticipate that participants will acquire knowledge and skills and will receive support needed to improve their capacity for managing HIV risk. Benefits to society in general are anticipated through the dissemination of intervention findings and community trainings in the ESTEEM treatment approach, if it is found to be efficacious. Results will better inform local and national public health agencies about potentially effective outreach and prevention strategies that can be delivered to young gay and bisexual men who experience lifetime stress-sensitive mental health disorders, such as depression and anxiety, and HIV-risk behavior. In sum, the potential benefits outweigh the potential risks to subjects.

The principal investigator will be responsible for monitoring the data and assuring protocol compliance. This protocol presents minimal risks to participants; unanticipated problems involving risks to subjects or others, including adverse events, are expected to be infrequent. In the event that such events occur, reportable events (which are events that are serious or life-threatening and unanticipated; or anticipated but occurring with a greater frequency than expected; and possibly, probably, or definitely related to study participation) or unanticipated problems involving risks to subjects or others that may require a temporary or permanent interruption of study activities will be reported immediately (if possible; if not, as soon as is possible), followed by a written report within five calendar days of the principal investigator becoming aware of the event to the institutional review board (IRB) and any appropriate funding and regulatory agencies. The principal investigator will apprise fellow investigators and study personnel of all unanticipated problems and adverse events that occur during the conduct of this research project (e.g., through regular study meetings, via email as they are reviewed by the principal investigator.). The protocol’s data safety monitoring board (DSMB) will also be informed of serious or unanticipated adverse events. The principal investigator, the IRB, or the DSMB have the authority to stop or suspend the study or require modifications.

## Discussion

This protocol describes a three-arm randomized control trial testing a transdiagnostic (cross-cutting) CBT intervention, called ESTEEM (Effective Skills to Empower Effective Men), that addresses the pathways through which minority stress compromises young gay and bisexual men’s co-occurring mental (e.g., depression), behavioral (e.g., substance use), and sexual (e.g., condomless anal sex) health problems. Young gay and bisexual men represent the largest group of individuals infected with HIV in the U.S. and one of the only risk groups in the U.S. for which new HIV infection rates are increasing. By addressing key sources of HIV risk among gay and bisexual men, including stigma-related stress and associated mental health and substance use difficulties, the intervention developed in this project has the potential to reduce HIV-risk behavior among young gay and bisexual men and therefore the spread of HIV.

To date, no randomized controlled trial has been conducted to determine the efficacy of mental health treatment for young gay and bisexual men, let alone the efficacy of a mental health treatment also capable of reducing behavioral risks such as substance use, condomless anal sex, and lack of PrEP initiation. We built ESTEEM upon a CBT platform designed to treat co-occurring mental health problems in the general population. This efficient transdiagnostic treatment approach, called the Unified Protocol, shows efficacy across mental and behavioral health problems in the general population [54], making it an ideal platform for intervening on young gay and bisexual men’s co-occurring mental (e.g., depression), behavioral (e.g., substance use), and sexual (e.g., condomless anal sex) risks. ESTEEM combines the CBT principles of the Unified Protocol with LGBTQ-affirmative principles drawn from community input during our formative interviews regarding the sources and experiences of minority stress in young gay and bisexual men’s lives. These LGBTQ-affirmative principles include (1) locating maladaptive behaviors in the context of their early and ongoing function, such as seeing depression and risk behaviors as learned minority stress reactions, (2) promoting young gay and bisexual men’s adaptive stigma coping using CBT skills, such as assertive communication, reducing avoidance, and emotion awareness/acceptance, (3) reworking minority stress cognitions such as internalized homophobia and rejection schemas, and (4) drawing on young gay and bisexual men’s personal resilience to build coping self-efficacy [37, 56].

Because of its transdiagnostic nature, ESTEEM could eliminate the need for numerous provider trainings and stand-alone treatments for separate problems. Our minority stress pathways approach to treating young gay and bisexual men’s co-occurring psychosocial problems is highly consistent with the Research Domain Criteria (RDoC) of the NIMH Strategic Plan [89]. The RDoC provides a comprehensive list of mechanisms that cut across psychosocial problems in the general population to spur a more focused, efficient search for transdiagnostic treatment targets. The RDoC mechanisms parallel the pathways in our model (Fig. 1) [37]. ESTEEM possesses promise for reducing the shared mechanisms linking minority stress with young gay and bisexual men’s health risks, paving the way for a unified treatment approach for the spectrum of young gay and bisexual men’s minority stress-driven health risks.

Delivering specific treatments for specific mental, behavioral, and sexual health problems represents the current state of evidence-based practice. However, dissemination and implementation of such treatments has not been ideal. A single intervention that reduces young gay and bisexual men’s depression, anxiety, substance use, and HIV risk by reducing the common minority stress pathways across these problems would represent an efficient, cost-effective alternative to currently isolated approaches, and holds great promise for reducing sexual orientation health disparities among young men.

## Data Availability

Not applicable.

## Abbreviations

BSI: Brief Symptom Inventory
CBT: Cognitive-behavioral therapy
CES-D: Center for Epidemiology Studies Depression Scale
CITI: Collaborative Institutional Training Initiative
CMHT: Community mental health treatment
*DSM*: *Diagnostic and Statistical Manual of Mental Disorders*
DSMB: Data safety monitoring board
ESTEEM: Effective Skills to Empower Effective Men
HAM-D: Hamilton Depression Rating Scale
HIV: Human immunodeficiency virus
*ICD*: *International Statistical Classification of Diseases and Related Health Problems*
IRB: Institutional review board
LGBT: Lesbian, gay, bisexual, transgender
LGBTQ: Lesbian, gay, bisexual, transgender, queer
MINI: Mini-International Neuropsychiatric Interview
NIH: National Institutes of Health
NIMH: National Institute of Mental Health
NYC: New York City
PrEP: Pre-exposure prophylaxis
PTSD: Post-traumatic stress disorder
RA: Research assistant
RCT: Randomized controlled trial
RDoC: Research Domain Criteria
SIP-AD: Short Inventory of Problems-Alcohol and Drugs
STI: Sexually transmitted infection
TLFB: Time-Line Follow-Back
US: United States
VCT: Voluntary HIV counseling and testing
